# Serum Sulfhydryl Groups, Malondialdehyde, Uric Acid, and Bilirubin as Predictors of Adverse Outcome in Heart Failure Patients due to Ischemic or Nonischemic Cardiomyopathy

**DOI:** 10.1155/2021/6693405

**Published:** 2021-04-15

**Authors:** Celina Wojciechowska, Wojciech Jacheć, Ewa Romuk, Anna Ciszek, Patryk Bodnar, Tomasz Chwalba, Martyna Waliczek, Mariusz Gąsior, Piotr Rozentryt

**Affiliations:** ^1^Second Department of Cardiology, Faculty of Medical Sciences in Zabrze, Medical University of Silesia, M. C. Skłodowskiej 10 Street, 41-800 Zabrze, Poland; ^2^Department of Biochemistry, Faculty of Medical Sciences in Zabrze, Medical University of Silesia, Jordana 19 Street, 41-808 Zabrze, Poland; ^3^Student Research Team at the Department of Cardiology, Faculty of Medical Sciences in Zabrze, Medical University of Silesia, M. C. Skłodowskiej 10 Street, 41-800 Zabrze, Poland; ^4^Department of Toxicology and Health Protection, Faculty of Health Sciences in Bytom, Medical University of Silesia, 41-902 Bytom, Poland; ^5^3rd Department of Cardiology, SMDZ in Zabrze, Medical University of Silesia, Silesian Centre for Heart Disease, 41-800 Zabrze, Poland

## Abstract

Oxidative stress plays a significant role in the pathogenesis of heart failure (HF). The aim of the study was to investigate the prognostic value of oxidation-reduction (redox) markers in patients with HF due to ischemic and nonischemic cardiomyopathy. The study included 707 patients of HF allocated into two groups depending on ethology: ischemic cardiomyopathy (ICM) (*n* = 435) and nonischemic cardiomyopathy (nICM) (*n* = 272), who were followed up for one year. The endpoint occurrence (mortality or heart transplantation) in a 1-year follow-up was similar in the ICM and nICM group. The predictive value of endpoint occurrence of oxidative stress biomarkers such as the serum protein sulfhydryl groups (PSH), malondialdehyde (MDA), uric acid (UA), bilirubin, and MDA/PSH ratio and other clinical and laboratory data were assessed in both groups (ICM and nICM) separately using univariate and multivariate Cox regression analyses. In multivariate analysis, the higher concentrations of UA (*p* = 0.015, HR = 1.024, 95% CI (1.005-1.044)) and MDA (*p* = 0.004, HR = 2.202, 95% CI (1.296-3.741)) were significantly associated with adverse prognosis in patients with ICM. Contrastingly, in patients with nICM, we observed that higher bilirubin concentration (*p* = 0.026, HR = 1.034, 95% CI (1.004-1.064)) and MDA/PSH ratio (*p* = 0.034, HR = 3.360, 95% CI (1.096-10.302)) were significantly associated with increased risk of death or HT. The results showed the association of different oxidative biomarkers on the unfavorable course of heart failure depending on etiology.

## 1. Introduction

The prognosis of patients with heart failure with reduced left ventricular ejection fraction is particularly poor. Dysregulated reduction-oxidation status, along with neurohormonal abnormalities and inflammation, is one of the common drivers of disease progression [1]. Increased formation of reactive oxygen species, a by-product of reduction-oxidation reactions, can lead to lipid peroxidation, protein carboxylation, cytoskeletal disruption, and DNA damage. Many clinical and laboratory parameters reflecting disturbed pathophysiological pathways in heart failure are used to identify patients at higher risk of unfavorable prognosis. Due to the extremely short half-life of ROS, it is not feasible to assess it directly. The compromised choice may be the assessment of stable by-products, modified under oxidative conditions associated with elevated ROS formation, which have been released into the circulation. Malondialdehyde (MDA) is a low molecular weight aldehyde that is formed by free radical attacks on polyunsaturated fatty acids. MDA testing as a biomarker of oxidative damage was useful as a favorable prognostic tool, e.g., sepsis or nephrotic syndrome [2, 3]. Protein sulfhydryl groups (PSH) predominate in serum in contrast to the intracellular space, which mainly consists of low molecular weight thiols [4]. Since reduced thiols are readily oxidized by reactive oxygen species, once oxidized, the thiols are less readily reduced in serum compared to their intracellular counterparts. Thus, free thiol depletion in serum reflects relatively stable systemic redox status. Recently published data revealed a prognostic role of serum free thiols in the general population [5]. The risk assessment in the outpatient population, despite stability of symptoms, is important to escalate the therapy in the appropriate time. The data analyzing the dependence prognosis on ischemic or nonischemic cardiomyopathy causes of heart failure come from many years ago [6]. In the Studies of Left Ventricular Dysfunction (SOLVD) including patients with heart failure, the etiology of the disease (ischemic or nonischemic) did not influence mortality in a one-year follow-up [7]. However, in most studies, the ischemic group had poorer prognosis [8, 9].

The mechanisms of oxidative stress and antioxidant defense are partially different in the ICM and the nICM patients [10]. Therefore, the aim of this study was to examine the prognostic value of clinical factors, oxidation-reduction (redox) biomarkers with special consideration of MDA and PSH, in two large cohorts of HF patients depending on ischemic or/and nonischemic etiology in a 1-year follow-up.

## 2. Study Group and Methods

### 2.1. The Inpatient

A clinic cohort with reduced ejection fraction heart failure (HFrEF) and chronic heart failure symptoms was considered potential candidates for heart transplantation and follow-up in the Prospective Registry of Heart Failure since 2003. Patients were stable and received optimal medical pharmacotherapy according to contemporary guidelines for at least 3 months before inclusion. Exclusion criteria were as follows: inability or unwillingness to provide informed consent, alcohol abuse or known antioxidant supplementation, and noncardiac diseases that affect life expectancy as judged by the treating physician. The data of 707 participants who had completed clinical laboratory assessment were included into the final analysis.

### 2.2. Endpoint of the Study

The endpoint of the study was all-cause mortality or urgent heart transplantation.

### 2.3. Clinical Assessments

Patients were characterized as an ischemic or nonischemic cardiomyopathy group according to the definition proposed by Felker et al. [11]. Besides any history of myocardial infarction or coronary revascularization, the basis for the qualification was the results of angiography performed within the last six months. At the time of study entry, detailed clinical data were obtained using a questionnaire. History of smoking was defined as current or previous use of tobacco products. Comorbidities such as hypertension, diabetes mellitus, or hypercholesterolemia were recognized based on actual measurements of respective variables, current medication, and clinical history. The body mass index (BMI) was calculated from mass and height measured on the day of inclusion visit. The functional capacity was assessed by NYHA classification and exercise maximum O_2_ uptake in the cardiopulmonary testing (MaxVO_2_) [12]. Two-dimensional transthoracic echocardiography was performed in all patients, and echocardiographic parameters were acquired in standard views as in the recommendation of the American Society of Echocardiography Committee [13]. A follow-up on patients was obtained via direct or phone contact with patients or their family every 6 months by a research personnel. For some patients not contacted through this mechanism, the exact data of death were obtained from the national identification number database. Prior to enrolment in the study, all participants provided written informed consent.

### 2.4. Biochemical Methods

Venous blood samples were obtained from each patient at the enrollment process. Each serum sample was separated by centrifugation at 1500 g for 10 minutes at 4°C. UA, bilirubin, lipid parameters, blood hemoglobin, and serum iron, sodium, creatinine, glucose, and albumin concentrations were measured with the use of the colorimetric method (Roche, Cobas 6000 e 501). The chemiluminescence method was used to determine NT-proBNP concentration (Roche, Cobas 6000 e 501). The serum for determining the oxidant parameters was frozen at -70°C until assayed. Total oxidant status (TOS) was measured by a spectrophotometric method by Erel. This method is based on the oxidation of Fe^2+^ ions to form Fe^3+^ in acidic environment and consists of measuring the color intensity of Fe^3+^ ion complexes with xylenol orange. The measurements were performed using EM280 biochemical analyzer. TOS was expressed in mmol/L. [14]. TAC was measured by Erel's colorimetric methods with the use of 2,2′-azino-bis (3-ethylbenzothiazoline-6-sulfonate) (ABTS+) [15]. In this reaction, reduced ABTS, a colorless molecule, is oxidized to blue-green ABTS+. The oxidized form of ABTS is reduced to the original colorless reduced form as a result of reactions with oxidizable substances. TAC was expressed in mmol/L. The sulfhydryl groups (PSH) were determined with the use of 5,5′-dithiobis (2-nitrobenzoic acid) (DTNB) in the method described by Koster. In this method, DTNB, after reduction by the sulfhydryl group-containing compounds, forms a yellow-colored anionic 5-thio-2-nitrobenzoic acid. The absorbance was measured with a Shimadzu 1700 UVVIS spectrophotometer at a wavelength of 412 nm [16]. PSH concentration was expressed in *μ*mol/g protein. Malondialdehyde (MDA) was measured by Ohkawa et al.'s spectrofluorimetric method. In this method, lipid peroxides react with thiobarbituric acid at the excitation wavelength 515 nm and emission wavelength 552 nm. The standard curve, prepared for 1,1,3,3-tetraethoxypropane, was used to calculate MDA concentration in *μ*mol/L [17].

### 2.5. Statistical Analysis

The subjects were allocated into groups according to the etiology of cardiomyopathy: ischemic (ICM) and nonischemic (nICM). Then, for the purposes of the analysis, in each group, the following patients were distinguished: the patient who achieved the endpoint (EP+) or without the endpoint (EP-) during one year of observation. Categorical data were displayed as proportions and were compared using the chi-square test with the Yates correction. Distribution of all continuous variables was evaluated by the Shapiro-Wilk test. Because of abnormal distribution of most continuous variables, the continuous data were presented as median with the first and third quartile. Data were tested with the Kruskal-Wallis ANOVA test; next, we assessed the differences between the selected groups using the nonparametric Mann Whitney *U* test and the chi^2^ test.

The estimation of risk of death and urgent transplantation was performed using a Cox proportional hazards model for the ICM and nICM group. All demographic, clinical, echocardiography, and laboratory variables and also medication data presented in Tables 1 and 2 were included in a univariate Cox analysis. A multivariate model was constructed separately for both groups based on the variables significantly associated with EP occurrence in each group (univariate analysis, *p* < 0.05). The results of the Cox analysis were reported as relative risks with corresponding 95% confidence intervals (CI). Cumulative survival over one year of follow-up was displayed using the Kaplan-Meier method, with comparison between groups depending on the cutoff value of concentrations of MDA, UA, and bilirubin or the MDA/PSH ratio calculated in Receiver Operation Characteristic (ROC) analysis, and the differences were tested for significance by the log-rank test. Results were considered statistically significant if *p* < 0.05. Statistical analysis was performed using Statistica 13.1 PL (TIBCO, Cracow, Poland).

## 3. Results

### 3.1. Baseline Characteristics of Subgroups in Relation to Etiology and Endpoint

In general, ischemic cardiomyopathy was more common than nonischemic cardiomyopathy (61.5% versus 38.5%). In the group of 435 patients of ICM etiology, 78 (17.9%) deaths and HT occurred over a one-year follow-up. 44 (16.2%) patients achieved an endpoint in 272 patients of the nICM group (chi^2^, *p* = 0.678). Baseline characteristics for patients enrolled in the study are shown in Table 1. Patients of the ICM group were older than those of the nICM group, but there were no differences between EP+ and EP-.

The percentage of patients in NYHA class III-IV was greater, and patients with implanted ICD were lower in EP+ groups in the ICM and nICM population. In both ICM and nICM, EP+ groups characterized a more enlarged left ventricle in echocardiography and maximum VO_2_ consumption in cardiopulmonary exercise testing and higher concentration of creatinine and NT-proBNP. In patients with ICM (EP+), lower ejection fraction in echocardiography, iron concentration, and higher HDL-cholesterol were observed.

The differences in redooxidative parameters are presented in Table 2. In patients with ICM (EP+), higher uric acid, bilirubin, and MDA concentrations and MDA/PSH ratio than those in patients with ICM (EP-) were indicated. Similarly, bilirubin and MDA concentrations and the MDA/PSH ratio but not uric acid were higher in the nICM (EP+) than in the nICM (EP-) group. Additionally, TAC was the most increased in nICM (EP+).

### 3.2. Association between Redox Reaction Parameters and Risk of Endpoint

#### 3.2.1. Uni- and Multivariate Cox Regression Analyses

Demographic and clinical data, laboratory results, comorbidities, pharmacotherapy, and oxidative stress-related parameters were assessed as risk factors for death or urgent heart transplantation in a 1-year follow-up in uni- and multivariate Cox regression analyses as presented in Tables 3 and 4. In univariate Cox regression analysis, higher levels of uric acid, bilirubin, MDA, and MDA/PSA were associated with endpoint occurrence in ICM patients. All assessed oxidative stress biomarkers without PSH in univariate Cox regression analysis were associated with the risk of death and HT in nICM.

In order to evaluate oxidative stress parameters in the context of confounders, a final multivariate model was calculated including all significant clinical, laboratory, and medication predictors significant in univariate analysis. After adjusting for significant predictors, only maxVO_2_ uptake in the cardiopulmonary testing, left ventricle ejection fraction, ICD presence, higher uric acid concentration (increase of risk (IoR) by 2.4% per 10 *μ*mol/L, *p* = 0.015), and higher serum MDA concentration (IoR by 220% per mmol/L, *p* = 0.004) were independent risk factors for death or heart transplantation in ICM. On the other hand, NT-proBNP, left ventricle ejection fraction, ICD presence, higher bilirubin concentration (increase of risk (IoR) by 3.4% per 1 *μ*mol/L, *p* = 0.026), and higher MDA/PSH ratio (IoR by 336%, *p* = 0.034) were independently related to endpoint occurrence in nICM.

**Figure 1:**
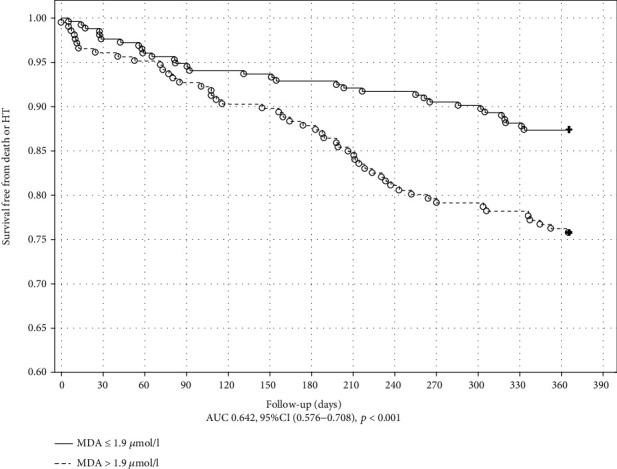
Kaplan-Meier curve of survival for the ICM group stratified by the MDA cutoff value of 1.9 *μ*mol/L. Log rank test (*p* = 0.034).

**Figure 2:**
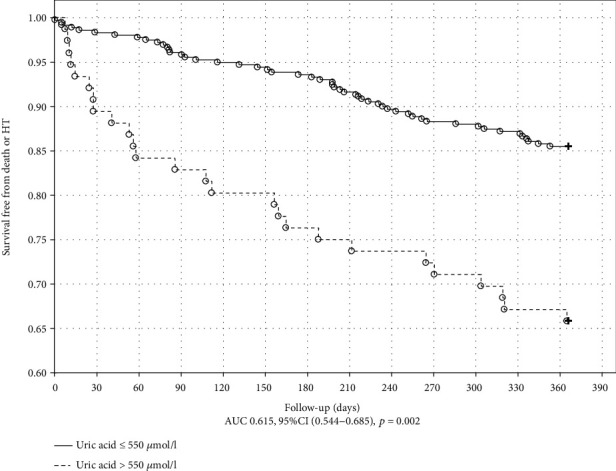
Kaplan-Meier curve of survival for the ICM group stratified by the uric acid cutoff value of 550 *μ*mol/L. Log rank test (*p* < 0.001).

**Figure 3:**
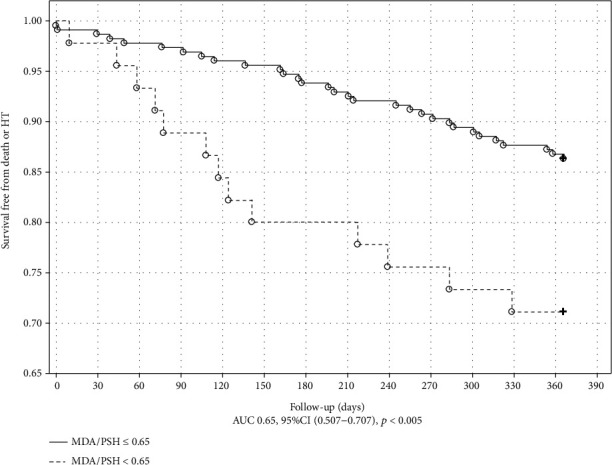
Kaplan-Meier curve of survival for the nICM group stratified by the MDA/PSH cutoff value of 0.65. Log rank test (*p* = 0.032).

**Figure 4:**
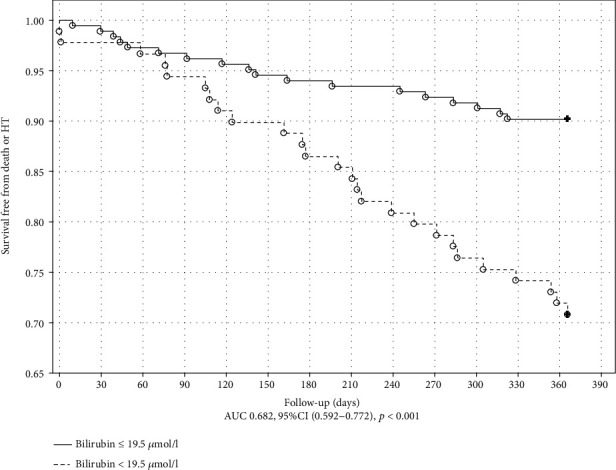
Kaplan-Meier curve of survival for the nICM group stratified by the bilirubin cutoff value of 19.5 *μ*mol/L. Log rank test (*p* = 0.003).

The Kaplan-Meier plot with a log-rank test for the outcome in ICM is shown in Figures 1 and 2. MDA and UA concentrations above the cutoff value obtained in ROC analysis were associated with poor outcome in ICM (*p* = 0.034 and *p* < 0.001, respectively). The Kaplan-Meier plot with a log-rank test for the endpoint occurrence in nICM is shown in Figures 3 and 4. The MDA/PSH ratio (*p* = 0.032) and bilirubin concentration (*p* = 0.003) above the cutoff value were related to risk of death and HT.

## 4. Discussion

While many clinical and biochemical variables have not only diagnostic but also prognostic value in heart failure, the question is whether these variables are of similar value in patients with ischemic and nonischemic heart failure. The observational studies, randomized clinical trials, and retrospective analyses of hospital records revealed inconsistent data on the impact of the etiology of heart failure on prognosis [7–9, 18].

In most of reports, a higher risk of death in patients with ICM compared with patients with nICM was indicated. In our study, the endpoint occurrence (all-cause mortality and HT) in a one-year follow-up was similar in ICM and nICM groups. A previous study has shown that oxidative stress is related to the severity of heart failure, although the results have been different in patients with ischemic and nonischemic cardiomyopathy [10]. According to our knowledge, this is the first study analyzing the influence of the reduction-oxidative balance parameters on the prognosis in patients with systolic heart failure, taking into account ischemic and nonischemic etiology. Independent redox biomarkers related to increased all-cause mortality and heart transplantation in a one-year follow-up were higher concentrations of uric acid and MDA in ICM. In contrast, higher levels of bilirubin and MDA/PSH were predictors of the endpoint in the nICM group. Surprisingly, NT-proBNP was a predictor only in the nICM and the maxVO_2_ and left ventricle ejection fraction only in the ICM group.

The results of our study showed that the sex and age of the patients did not affect the achievement of the one-year endpoint in both groups ICM and nICM. Anker et al. indicated that uric acid, age, and left ventricle ejection fraction, but not peak VO_2_, were predictive of impaired survival in European heart failure patients (60% of ischemic etiology) [19]. Similarly, UA was a prognostic marker of all-cause mortality after adjustment to NYHA class and creatinine clearance in the population of heart failure patients (70% of ischemic etiology) as described by Jankowska et al. [20]. Sakai et al. confirmed the important prognostic role of UA in a Japanese population. Both increased UA and BNP were associated with mortality independently from etiology, sex, age, NYHA class, and LVEF [21]. Hyperuricemia may reflect raised xanthine oxidase activity in heart failure; this enzyme system is an important source of oxygen free radicals [22, 23]. Uric acid concentration is affected by using a xanthine oxidase inhibitor (allopurinol). Allopurinol was used more often in the nICM groups (EP+ and EP-) than, respectively, in the ICM groups (EP+ and EP-). The median uric acid concentration did not differ statistically between ICM and nICM; however, uric acid concentration was statistically significantly higher in the ICM EP+ than in ICM EP- (*p* < 0.002). Higher uric acid level was a marker associated with increased mortality in this group;

Our results indicating malondialdehyde, a by-product of polyunsaturated fatty acid lipid peroxidation, as a predictor of death and heart transplantation only in ICM, are consistent with observation published by Radovanovic et al. that malondialdehyde predicted mortality in patients with chronic ischemic heart failure during 13-month observation [24]. The previous reports demonstrated increase in MDA concentration in patients with HF of different etiologies, but the relationship between MDA and the severity of heart failure assessed as NYHA class, exercise intolerance LVEF, and invasive hemodynamic measurements is inconsistent [25–30]. The last papers do not confirm such a relationship perhaps due to the use of standardized treatment with inhibitors of the renin-angiotensin system and beta-blockers [29, 30]. MDA, the highest quartile concentration, was an independent risk factor for a cardiovascular event (myocardial infarction—fatal, nonfatal, stroke, and unstable angina) as compared to the lowest quartile. The prognostic value was independent of interleukin-6, C-reactive protein, and classical risk factors for atherosclerosis [31]. The above observations draw our attention to the lipid profile of patients with ICM. In lipid parameters, it was surprising that only the high concentration of HDL cholesterol in univariable analysis had a protective effect on survival in the ischemic group. However, the ICM EP+ group had a significantly higher LDL than the nICM EP+ group despite more frequent use of statins. It should be emphasized that patients were enrolled in the study before the current very strict LDL target recommendations [32]. This fact may confirm the large role of LDL in the pathogenesis of coronary diseases in general. LDL cholesterol is oxidized by MDA to MDA-LDL molecules. These combinations have been shown to be particularly atherogenic [33, 34]. Tani et al. proved that the concentration of MDA-LDL is independent of the overall LDL. It may depend on the oxidative state of a patient individually. Their study showed that patients with coronary artery disease have increased levels of MDA-LDL. It has been suggested that this may be a promising indicator in assessing the risk of coronary artery disease [35].

In the ICM group, the PSH, MDA/PSH index, and NT-proBNP in multivariable Cox regression analysis indices showed no prognostic significance in predicting the endpoint. The situation is different in the case of patients with nICM, in whom the MDA/PSH ratio and NT-proBNP were indicated as risk factors for death or OHT. Increasing attention, for the last years, has been paid to the role of oxidative stress in CVD, since it may lead to endothelial dysfunction [36]. Frenay et al. show that high serum concentrations of PSH are associated with a favorable cardiovascular risk profile and better survival for patients with the transplanted kidney [37]. In acute myocardial infarction, it was shown that thiols are an oxidative stress marker [38] and that plasma thiols are significantly lower in subjects with congestive heart failure, as compared to healthy subjects [39]. Recently, Abdulle et al. found that protein-adjusted serum free thiol concentrations were able to predict the risk of all-cause mortality and cardiovascular events after adjustment for confounders in the general population. In the subgroup of subjects with a known history of cardiovascular disease (*n* = 217), regression analyses revealed age- and sex-adjusted associations between protein-adjusted serum free thiol concentrations and all-cause mortality when comparing the upper two tertiles with the lowest tertile and lack of significant associations with the risk of cardiovascular events [5]. In the study by Koning et al., they found in a group of 101 patients with stable heart failure that free serum thiols are associated independently with age, cholesterol, and parathyroid hormone. Free thiols and age were inversely related. In multivariable analysis, an association of PSH with NT-proBNP was not identified. Both serum free thiols per gram of protein below the mean and NT-proBNP above the median were predictors of a composite endpoint: HF-related rehospitalisation and all-cause mortality. However, further analysis adjusting PSH to establish prognostic factors in HF (age, eGFR, and NT-proBNP) revealed no longer significant association with the endpoint [40]. 72% of patients in this study characterized ischemic etiology. The participants were older compared to our nICM group and had less reduced ejection fraction. Despite free thiols (PSH, sulfhydryl groups) are more accurately reflecting the systemic in vivo redox status as compared to many other individual oxidant or antioxidant factors and their derivatives [41], in our study, the MDA/PSH ratio but not PSH was a valuable predictor of adverse outcome in nICM.

The positive association of reduced thiol concentration with increased circulating bilirubin was indicated in unconjugated hyperbilirubinemia (Gilbert syndrome). The modulation by bilirubin lipid status may be the explanation of protection from ischemic heart disease in Gilbert syndrome [42]. The epidemiological studies indicate that mildly elevated bilirubin concentrations were protective against cardiovascular disease and all-cause mortality [43–45]. However, we did not observe significance of bilirubin concentration with endpoint prediction in heart failure of ischemic etiology. Moreover, higher bilirubin was associated with adverse outcome in the nICM group.

It is disappointing to find that using allopurinol did not improve patients' prognosis in any group of our study, although previous studies indicated that allopurinol may have the ability to decrease mortality in HF. [46, 47]. The explanation may be that the dose was too low, because higher doses of allopurinol have a greater impact on cardiovascular outcomes than lower doses [47, 48]. Oxidation-reduction systems play an important role in the progression of heart failure. It has been proven that some of the commonly used drugs in HF, such as ACE inhibitors or beta-blockers, affect this system [49, 50]. Pharmacological treatment of HF recommended by guidelines has progressed over the past decades and has improved the prognosis in this group of patients. Recently, the importance of personalizing treatment has been emphasized because the population enrolled to the randomized trials does not always represent the patients we treat. In most HF clinical trials, patients predominantly were male with ischemic etiology. Thus, we decided for separate analysis of each group and we indicated that only the ICD presence was a common prognostic factor for both groups. The others (max VO_2_, LVEF, uric acid, and MDA) were risk factors for the endpoint in ischemic etiology whereas thiazide diuretic therapy, concentration of NT-proBNP, bilirubin, and MDA/PSH ratio in the nonischemic group. Our results proved influence of different redox biomarkers on one-year prognosis in HF cohorts of ischemic and nonischemic etiology. Nowadays, it is usually difficult to predict which patients will benefit most from the different therapy; maybe when we pay attention on deviations in redox biomarkers, it would help us in qualifying patients for the use of, e.g., allopurinol, as donors of SH groups.

## Figures and Tables

**Table 1:** Baseline characteristic of the examined group and comparison of subgroups separated on the basis of prognosis.

	ICM			nICM		C vs. D, *p*	A vs. C, *p*	B vs. D, *p*	ANOVA (A-D)
	A (*n* = 78), death or HT	B (*n* = 357), without endpoint	A vs. B, *p*	C (*n* = 44), death or HT	D (*n* = 228), without endpoint				
General characteristics, median (25^th^-75^th^ percentiles) and ^∗^*n* (%)									
Male^∗^	65 (83.33)	308 (86.27)	0.621	38 (86.36)	191 (87.77)	0.837	0.855	0.475	0.813
Age (years)	56.50 (52.00-1.00)	56.00 (52.00-61.00)	0.958	50.00 (43.00-57.50)	49.00 (38.00-55.50)	0.278	<0.001	0.129	<0.001
BMI (kg/m^2^)	26.05 (22.09-29.07)	26.45 (24.14-29.30)	0.142	23.44 (20.89-26.43)	26.07 (22.70-29.33)	0.007	0.030	0.285	<0.001
Duration of symptoms (months)	40.58 (13.50-70.43)	33.30 (14.13-64.73)	0.402	59.03 (23.38-86.45)	29.57 (9.58-66.57)	0.004	0.083	0.730	0.018
NYHA class (III-IV)^∗^	65 (83.33)	181 (50.70)	<0.001	39 (88.64)	115 (50.44)	<0.001	0.598	0.982	<0.001
T6M (m)	320.0 (233.0-363.0)	378.0 (312.0-407.5)	0.004	346.5 (291.0-409.0)	401.0 (341.0-454.0)	0.090	0.169	0.009	<0.001
Max VO_2_ (mL/min/kg)	12.30 (9.80-14.00)	15.20 (12.40-18.70)	<0.001	11.90 (9.70-14.10)	16.00 (13.30-20.10)	<0.001	0.878	0.135	<0.001
LVEDD (mm)	70.00 (65.00-76.00)	68.00 (63.00-75.00)	0.054	73.00 (63.00-78.0)	70.00 (65.00-77.00)	0.525	0.605	0.000	0.059
LVEDV (mL)	232.5 (195.0-314.0)	204.0 (156.0-264.0)	0.002	263.8 (222.0-340.0)	236.0 (179.0-307.0)	0.038	0.041	0.011	<0.001
LVEF (%)	19.00 (16.00-23.00)	25.00 (20.00-33.00)	<0.001	22.50 (17.00-26.00)	23.50 (20.00-29.00)	0.063	0.069	0.034	<0.001
Basic biochemistry, median (25^th^-75^th^ percentiles)									
Hemoglobin (g/dL)	14.02 (12.89-14.99)	13.86 (13.05-14.83)	0.792	14.10 (12.81-15.39)	14.18 (13.14-15.29)	0.892	0.329	0.010	0.192
Iron (*μ*mol/L)	14.85 (10.80-19.04)	17.33 (12.38-21.90)	0.005	16.35 (10.40-22.75)	17.95 (12.35-23.60)	0.287	0.301	0.500	0.013
Creatinine (*μ*mol/L)	95.00 (81.0-123.0)	85.0 (72.00-104.0)	0.003	87.50 (78.00-114.5)	81.00 (70.00-97.00)	0.016	0.790	0.149	<0.001
Serum protein (g/L)	72.00 (67.00-76.00)	71.00 (66.00-75.00)	0.224	72.50 (65.00-78.50)	71.00 (67.00-75.00)	0.380	0.577	0.114	0.457
Albumin (g/L)	40.00 (37.00-44.00)	42.00 (39.00-44.00)	0.048	41.00 (38.00-44.00)	42.00 (40.00-45.00)	0.110	0.155	0.123	0.034
Fasting glucose (mmol/L)	5.70 (5.00-6.80)	5.60 (5.00-6.20)	0.227	5.30 (4.95-5.95)	5.50 (4.90-6.10)	0.879	0.226	0.597	0.127
Cholesterol (mmol/L)	4.26 (3.66-5.11)	4.24 (3.65-5.22)	0.576	4.21 (3.35-5.15)	4.35 (3.62-5.20)	0.344	0.110	0.252	0.705
Triglycerides (mmol/L)	1.16 (0.89-1.63)	1.20 (0.84-1.75)	0.675	1.11 (0.76-1.41)	1.25 (0.93-1.75)	0.022	0.678	0.523	0.134
HDL (mmol/L)	1.08 (0.83-1.35)	1.16 (0.97-1.42)	0.012	1.16 (0.79-1.42)	1.13 (0.93-1.39)	0.512	0.780	0.362	0.064
LDL (mmol/L)	2.52 (1.92-3.18)	2.34 (1.86-3.09)	0.505	2.31 (1.75-3.23)	2.49 (1.93-3.12)	0.854	0.012	0.129	0.878
NT-proBNP (pg/mL/100)	28.50 (15.04-48.10)	11.70 (5.49-23.99)	<0.001	47.24 (22.16-87.99)	13.63 (6.20-28.11)	<0.001	<0.001	0.285	<0.001
Comorbidities, *n* (%)									
Diabetes	35 (44.87)	111 (31.09)	0.28	11 (25.00)	43 (18.86)	0.466	0.048	0.002	<0.001
Arterial hypertension	42 (53.85)	221 (61.90)	0.946	20 (45.45)	106 (46.49)	0.969	0.483	0.001	0.002
Atrial fibrillation	17 (21.79)	58 (16.25)	0.313	16 (36.36)	73 (32.02)	0.072	0.127	0.043	<0.001
ICD presence	3 (3.85)	104 (29.13)	0.001	3 (6.82)	91 (39.91)	0.001	0.770	0.009	<0.001
Smoker	21 (26.92)	112 (31.37)	0.001	15 (34.09)	96 (42.11)	0.411	0.531	0.011	0.025
Treatment, *n* (%)									
Beta-blockers	77 (98.72)	352 (98.60)	0.650	41 (93.18)	223 (97.81)	0.240	0.942	0.694	0.104
ACE inhibitors, *n* (%)	63 (80.77)	317 (88.80)	0.081	31 (70.45)	199 (87.28)	0.009	0.282	0.673	0.004
ARB	5 (6.41)	38 (10.64)	0.100	5 (11.36)	25 (10.96)	0.853	0.539	0.988	0.687
Loop diuretics, *n* (%)	75 (96.15)	285 (79.83)	0.001	43 (97.73)	211 (92.54)	0.350	0.952	0.001	<0.001
Thiazide diuretics, *n* (%)	12 (15.38)	37 (10.36)	0.283	15 (34.09)	24 (10.53)	0.001	0.031	0.939	<0.001
MRA	73 (93.59)	319 (89.36)	0.355	39 (88.64)	219 (96.11)	0.096	0.539	0.006	0.017
Statins	55 (70.51)	274 (76.75)	0.010	17 (38.64)	120 (52.63)	0.125	0.001	0.001	<0.001
Fibrates	2 (2.56)	15 (4.20)	0.724	0 (0.00)	9 (3.95)	0.379	0.777	0.845	0.518
Digitalis	40 (51.28)	140 (39.22)	0.067	27 (61.36)	117 (51.32)	0.290	0.376	0.005	0.003
XO blockers	27 (34.62)	102 (28.57)	0.357	25 (56.82)	116 (57.14)	0.577	0.029	<0.001	<0.001

BMI: body mass index; NYHA: New York Heart Association functional class; max VO_2_: maximum oxygen uptake; LVEDD: left ventricle end-diastolic diameter; LVEDV: left ventricle end-diastolic volume; LVEF: left ventricle ejection fraction; NT-proBNP: N-terminal pro-B-type natriuretic peptide; ICD: implantable cardioverter defibrillator; ACE inhibitor: angiotensin-converting enzyme inhibitor; ARB: angiotensin receptor blocker; MRA: mineralocorticoid receptor antagonists; NS: nonsignificant; XO: xanthine oxidase; A: ICM patients (EP+); B: ICM patients (EP-); C: nICM patients (EP+); D: nICM patients (EP-).

**Table 2:** Redox parameters of the examined group and comparison of subgroups separated on the basis of prognosis (median (25^th^-75^th^ percentiles)).

	ICM			nICM		C vs. D, *p*	A vs. C, *p*	B vs. D, *p*	ANOVA (A-D)
	A (*n* = 78), death or HT	B (*n* = 357), without endpoint	A vs. B, *p*	C (*n* = 44), death or HT	D (*n* = 228), without endpoint				
TAC (mmol/L)	1.13 (1.02-1.27)	1.14 (1.040-1.25)	0.861	1.140 (1.060-1.250)	1.08 (0.98-1.21)	0.017	0.759	<0.001	<0.001
TOS (mmol/L)	5.15 (4.10-6.60)	4.70 (4.000-5.90)	0.227	5.40 (4.45-6.60)	5.10 (4.20-6.05)	0.105	0.298	0.126	0.069
Uric acid (*μ*mol/L)	44.55 (36.20-59.90)	39.90 (32.30-49.30)	0.002	42.35 (35.90-56.95)	40.80 (31.40-48.45)	0.068	0.699	0.642	0.002
Bilirubin (*μ*moL/L)	14.80 (10.10-24.70)	12.30 (9.10-18.60)	0.036	20.55 (13.80-33.00)	14.70 (10.00-20.60)	<0.001	0.007	0.014	<0.001
PSH (*μ*mol/g of protein)	3.85 (3.10-5.00)	4.10 (3.100-5.300)	0.592	4.45 (3.100-5.450)	4.80 (3.80-5.60)	0.146	0.317	<0.001	<0.001
MDA (*μ*mol/L)	2.00 (1.60-2.40)	1.80 (1.400-2.100)	<0.001	1.90 (1.500-2.250)	1.70 (1.30-2.00)	0.024	0.162	0.144	<0.001
MDA/PSH ratio	0.52 (0.36-0.74)	0.41 (0.308-0.622)	0.004	0.42 (0.291-0.705)	0.35 (0.26-0.50)	0.029	0.146	<0.001	<0.001

TAC: total antioxidant capacity; TOS: total oxidant status; MDA: malondialdehyde; PSH: sulfhydryl groups; A: ICM patients (EP+); B: ICM patients (EP-); C: nICM patients (EP+); D: nICM patients (EP-).

**Table 3:** Clinical and laboratory parameters as risk factors for death or OHT of patients with ICM in a 1-year follow-up. Uni- and multivariable Cox regression analysis.

	Univariable Cox regression analysis			Multivariable Cox regression analysis (complete data, *n* = 387)		
	*p*	HR	95% CI	*p*	HR	95% CI
General characteristics						
Female (yes/no)	0.721	1.113	0.618-2006			
Age (years)	0.861	0.998	0.972-1.024			
BMI (kg/m^2^)	0.134	0.959	0.908-1.013			
Duration of symptoms before inclusion (months)	0.233	1.003	0.998-1.007			
NYHA class (by one)	<0.001	2.396	1.774-3.237	0.559	1.143	0.730-1.798
Max VO_2_ (by 1 mL/min/kg)	<0.001	0.849	0.802-0.899	0.044	0.923	0.854-0.998
LVEDD (mm)	0.030	1.027	1.003-1.051			
LVEDV (mL)	<0.001	1.005	1.002-1.007			
LVEF (by 1%)	<0.001	0.868	0.835-0.903	<0.001	0.881	0.831-0.934
Basic biochemistry						
Hemoglobin (g/dL)	0.906	0.992	0.871-1.130			
Iron concentration (*μ*mol/L)	0.012	0.960	0.930-0.991	0.052	0.957	0.915-1.000
Creatinine (*μ*mol/L)	0.007	1.005	1.001-1.010	0.992	1.000	0.993-1.007
Serum protein (g/L)	0.226	1.021	0.987-1.056			
Albumin (g/L)	0.019	0.940	0.892-0.990	0.318	0.961	0.889-1.039
Fasting glucose (mmol/L)	0.060	1.098	0.996-1.212			
Total cholesterol (mmol/L)	0.738	0.970	0.814-1.157			
Triglycerides (mmol/L)	0.417	0.886	0.660-1.188			
Cholesterol HDL (mmol/L)	0.008	0.423	0.224-0.797	0.578	0.817	0.401-1.665
Cholesterol LDL (mmol/L)	0.369	1.094	0.899-1.332			
NT-proBNP (100 pg/mL)	<0.001	1.017	1.011-1.023	0.322	1.005	0.995-1.014
Comorbidities						
Diabetes (yes/no)	0.025	1.663	1.064-2.599	0.978	0.993	0.588-1.677
Arterial hypertension (yes/no)	0.196	0.745	0.477-1.163			
Atrial fibrillation (yes/no)	0.123	1.481	0.899-2.438			
ICD presence (yes/no)	<0.001	0.133	0.049-0.363	<0.001	0.130	0.040-0.422
Smoker (yes/no)	0.586	0.879	0.553-1.397			
Treatment						
Beta-blockers (yes/no)	0.840	1.226	0.171-8.801			
ACE inhibitors (yes/no)	0.033	0.542	0.308-0.951	0.493	0.795	0.412-1.532
ARB (yes/no)	0.467	0.751	0.347-1.625			
Loop diuretics (yes/no)	0.002	5.923	1.873-18.729	0.867	1.112	0.319-3.877
Thiazide diuretics (yes/no)	0.158	1.529	0.848-2.745			
MRA (yes/no)	0.166	1.893	0.767-4.667			
Statins (yes/no)	0.175	0.714	0.439-1.162			
Fibrates (yes/no)	0.500	0.617	0.152-2.509			
Digitalis (yes/no)	0.035	1.567	1.032-2.380	0.416	0.803	0.473-1.363
XO inhibitors (yes/no)	0.345	1.237	0.796-1.922			
Oxidative stress parameters						
TAC (mmol/L)	0.469	1.518	0.490-4.698			
TOS (mmol/L)	0.700	1.015	0.942-1.093			
OSI (TOS/TAC)	0.717	1.014	0.940-1.094			
Uric acid (10 *μ*mol/L)	<0.001	1.003	1.002-1.004	0.015	1.024	1.005-1.044
Bilirubin (*μ*mol/L)	<0.001	1.026	1.011-1.041	0.569	0.994	0.972-1.016
MDA (*μ*mol/L)	<0.001	2.125	1.493-3.023	0.004	2.202	1.296-3.741
MDA/PSH ratio	0.036	1.617	1.032-2.532	0.334	0.671	0.298-1.508

BMI: body mass index; NYHA: New York Heart Association functional class; max VO_2_: maximum oxygen uptake; LVEDD: left ventricle end-diastolic diameter; LVEDV: left ventricle end-diastolic volume; LVEF: left ventricle ejection fraction; NT-proBNP: N-terminal pro-B-type natriuretic peptide; ICD: implantable cardioverter defibrillator; ACE inhibitor: angiotensin-converting enzyme inhibitor; ARB: angiotensin receptor blocker; MRA: mineralocorticoid receptor antagonists; XO: xanthine oxidase; TAC: total antioxidant capacity; TOS: total oxidant status; MDA: malondialdehyde; PSH: sulfhydryl groups.

**Table 4:** Clinical and laboratory parameters as risk factors for death or OHT of patients with nICM in a 1-year follow-up. Uni- and multivariable Cox regression analysis.

	Univariable Cox regression analysis			Multivariable Cox regression analysis (complete data, *n* = 247)		
	*p*	HR	95% CI	*p*	HR	95% CI
General characteristics						
Male (yes/no)	0.699	1.184	0.502-2.793			
Age (years)	0.286	1.014	0.988-1.041			
BMI (kg/m^2^)	0.012	0.923	0.867-0.982	0.772	1.013	0.928-1.106
Duration of symptoms before inclusion (months)	0.033	1.005	1.001-1.009	0.179	1.005	0.998-1.012
NYHA class	<0.001	3.879	2.517-5.978	0.144	1.687	0.837-3.400
Maximum measured VO_2_ (by 1 mL/min/kg b.m.)	<0.001	0.826	0.762-0.896	0.121	0.919	0.825-1.023
LVEDD (mm)	0.714	0.995	0.969-1.022			
LVEDV (mL)	0.101	1.003	1.000-1.006			
LVEF (by 1%)	0.886	0.997	0.961-1.035			
Basic biochemistry						
Hemoglobin (mmol/L)	0.987	1.001	0.841-1.193			
Iron concentration (*μ*mol/L)	0.651	0.993	0.962-1.024			
Creatinine (*μ*mol/L)	0.039	1.008	1.000-1.016	0.703	0.997	0.982-1.012
Serum protein (g/L)	0.256	1.027	0.981-1.074			
Albumin (g/L)	0.053	0.938	0.879-1.001			
Fasting glucose (mmol/L)	0.491	1.059	0.899-1.248			
Total cholesterol (mmol/L)	0.918	0.988	0.778-1.254			
Triglycerides (mmol/L)	0.329	0.844	0.601-1.187			
Cholesterol HDL (mmol/L)	0.645	0.865	0.468-1.601			
Cholesterol LDL (mmol/L)	0.588	1.086	0.805-1.466			
NT-proBNP (100 pg/mL)	<0.001	1.021	1.015-1.027	<0.001	1.019	1.008-1.031
Comorbidities						
Diabetes (yes/no)	0.405	1.334	0.677-2.626			
Arterial hypertension (yes/no)	0.885	0.958	0.536-1.711			
Atrial fibrillation (yes/no)	0.586	1.181	0.649-2.149			
ICD presence (yes/no)	<0.001	0.080	0.019-0.331	0.009	0.180	0.050-0.645
Smoker (yes/no)	0.342	0.745	0.406-1.367			
Treatment						
Beta-blockers (yes/no)	0.037	0.287	0.089-0.926	0.895	0.838	0.060-11.629
ACE inhibitors (yes/no)	<0.001	0.334	0.178-0.627	0.446	0.657	0.223-1.935
ARB (yes/no)	0.662	1.211	0.513-2.856			
Loop diuretics (yes/no)	0.249	3.206	0.442-23.253			
Thiazide diuretics (yes/no)	<0.001	3.685	2.023-6.713	0.027	2.702	1.117-6.535
MRA (yes/no)	0.034	0.368	0.146-0.929	0.884	0.876	0.150-5.132
Statins (yes/no)	0.100	0.605	0.333-1.102			
Fibrates (yes/no)	0.526	0.527	0.073-3.823			
Digitalis (yes/no)	0.306	1.363	0.754-2.463			
XO inhibitors (yes/no)	0.767	0.992	0.939-1.048			
Oxidative stress parameters						
TAC (mmol/L)	0.019	5.454	1.325-22.444	0.547	0.334	0.009-11.841
TOS (mmol/L)	0.037	1.099	1.006-1.201	0.221	0.883	0.723-1.078
OSI (TOS/TAC)	0.590	1.019	0.953-1.089			
Uric acid (10 *μ*mol/L)	0.010	1.025	1.006-1.045	0.268	1.019	0.985-1.054
Bilirubin (*μ*moL/L)	<0.001	1.034	1.019-1.049	0.026	1.034	1.004-1.064
PSH (*μ*mol/g of protein)	0.146	0.851	0.685-1.058			
MDA (*μ*mol/L)	0.032	1.823	1.052-3.160	0.326	1.665	0.603-4.602
MDA/PSH ratio	<0.001	3.414	2.047-5.695	0.034	3.360	1.096-10.302

BMI: body mass index; NYHA: New York Heart Association functional class; max VO_2_: maximum oxygen uptake; LVEDD: left ventricle end-diastolic diameter; LVEDV: left ventricle end-diastolic volume; LVEF: left ventricle ejection fraction; NT-proBNP: N-terminal pro-B-type natriuretic peptide; ICD: implantable cardioverter defibrillator; ACE inhibitor: angiotensin-converting enzyme inhibitor; ARB: angiotensin receptor blocker; MRA: mineralocorticoid receptor antagonists; XO: xanthine oxidase; TAC: total antioxidant capacity; TOS: total oxidant status; MDA: malondialdehyde; PSH: sulfhydryl groups.

## Data Availability

The original data is available after contact with the corresponding author.
